# Microbial Metabolites of Flavan-3-Ols and Their Biological Activity

**DOI:** 10.3390/nu11102260

**Published:** 2019-09-20

**Authors:** Estefanía Márquez Campos, Peter Stehle, Marie-Christine Simon

**Affiliations:** 1 Department of Nutrition and Food Sciences, Nutrition and Microbiota, University of Bonn, 53115 Bonn, Germany; estefania.marquezc@gmail.com; 2 Department of Nutrition and Food Sciences, Nutritional Physiology, University of Bonn, 53115 Bonn, Germany; p.stehle@uni-bonn.de

**Keywords:** polyphenols, flavonoids, microbiota, metabolism

## Abstract

Flavan-3-ols are the main contributors to polyphenol intake. Many varying beneficial health effects in humans have been attributed to them, including the prevention of cardiovascular disease and cancer. Nevertheless, the mechanisms by which these flavonoids could exert beneficial functions are not entirely known. Several in vitro studies and in vivo animal models have tried to elucidate the role of the specific colonic metabolites on the health properties that are attributed to the parent compounds since a larger number of ingested flavan-3-ols reach the colon and undergo there microbial metabolism. Many new studies about this topic have been performed over the last few years and, to the best of our knowledge, no scientific literature review regarding the bioactivity of all identified microbial metabolites of flavan-3-ols has been recently published. Therefore, the aim of this review is to present the current status of knowledge on the potential health benefits of flavan-3-ol microbial metabolites in humans while using the latest evidence on their biological activity.

## 1. Introduction

**Figure 1:**
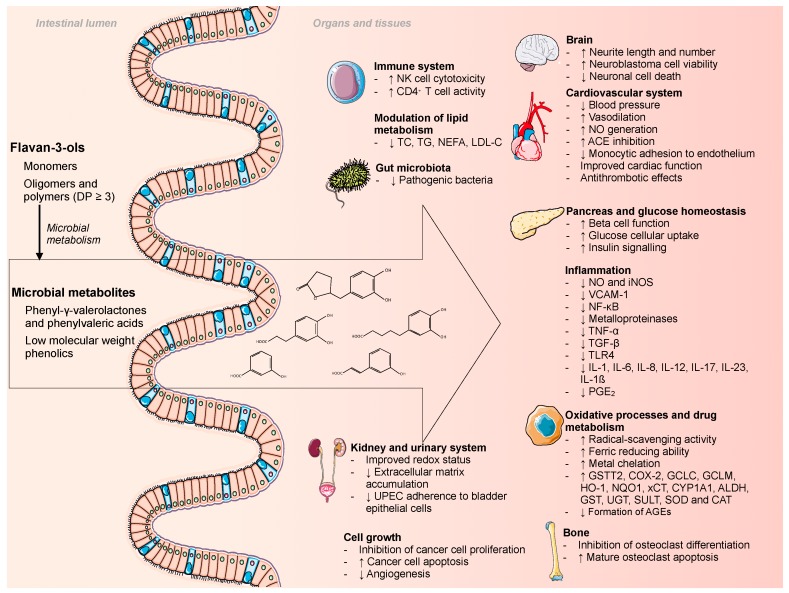
Schematic view of the postulated bioactivity of flavan-3-ol microbial metabolites. ACE: angiotensin I-converting enzyme; AGE: advanced glycation end-products; ALDH: aldehyde dehydrogenase; CAT: catalase; CD4⁺ cells: T helper cells; COX-2: cyclooxygenase-2; CYP1A1: cytochrome P450 1A1; DP: degree of polymerization; GCLC: glutamate-cysteine ligase catalytic subunit; GCLM: glutamate-cysteine ligase modifier subunit; GST: glutathione S-transferase; GSTT2: glutathione S-transferase theta-2; HO-1: heme oxygenase 1; IL: interleukin; iNOS: inducible nitric oxide synthase; LDL-C: low-density lipoprotein cholesterol; NEFA: non-esterified fatty acids; NF-κB: nuclear factor kappa-light-chain-enhancer of activated B cells; NK cell: natural killer cells; NO: nitric oxide; NQO1: NADH:quinone oxidoreductase-1; PGE_2_: prostaglandin E_2_; SOD: superoxide dismutase; SULT: sulfotransferase; TC: total cholesterol; TG: triglycerides; TLR-4: toll-like receptor 4; TNF: tumor necrosis factor; UGT: UDP-glucuronosyltransferase; UPEC: uropathogenic *Escherichia coli;* VCAM: vascular cell adhesion molecule; xCT: cystine/glutamate anti-porter.

Polyphenols exert numerous beneficial health effects in the human organism. The main contributors to polyphenol intake are flavan-3-ols [1,2], which constitute the most complex flavonoid subclass. After ingestion, flavan-3-ols can reach the colon in high proportions, where they can be transformed into microbial metabolites by the resident microbiota. These catabolites might be responsible for the health promoting effects that are attributed to the parent compounds (Figure 1).

Monagas et al. laboriously complied all of the available scientific information related to the metabolic pathway of flavan-3-ols in the human organism and reviewed the biological activities that were attributed to their phase-II and microbial metabolites [3]. However, since then, many new studies regarding this topic have been performed and, to the best of our knowledge, no other scientific literature review about the bioactivity of all of the identified microbial metabolites of flavan-3-ols to date has been recently published. Only Mena et al. recently addressed the bioactivity of phenyl-γ-valerolactones and phenylvaleric acids: two of the several groups of flavan-3-ol colonic metabolites [4]. Thus, the aim of this review is to summarize the most recent scientific publications on biological activity of microbial metabolites of flavan-3-ols to present the current status of knowledge on this topic.

## 2. Origin

Flavan-3-ols are present in food not only in the form of monomers, but also as oligomeric and polymeric proanthocyanidins, whose degree of polymerization can reach up to 50 units or more [5].

They are commonly found in foods, such as fruits, green tea, red wine, and chocolate [6], although the main dietary source in the population differs demographically and, therefore, the type of flavan-3-ol that is consumed also differs [7]. Their original content in food can also vary due to several factors, such as the plant variety, the time of harvest, environmental factors, processing, and storage conditions [6]. For this reason, flavan-3-ol content in foods has been detected at very different concentrations ranging, for example, between 6 and 544 mg/100 g green tea, or between 77 and 273 mg/100 g dark chocolate [7].

These flavonoids have been shown to exert beneficial health effects in humans in observational as well as in intervention studies. For example, their intake might prevent cardiovascular diseases [8] or cancer [9]. Nevertheless, the mechanisms by which they could have this positive impact on health are not entirely known. In fact, the bioavailability of flavan-3-ols might be lower than 4 % in humans [10].

Several factors could have an influence on bioavailability, such as food matrix, compound concentration, or gut microbiota composition. Additionally, the degree of polymerization influences their bioavailability: generally, monomeric flavan-3-ols can be absorbed in the small intestine, while the structures with a higher polymerization degree are metabolized by the intestinal microbiota before they are finally absorbed [3].

Therefore, the fact that a large proportion of flavan-3-ols reach the colon suggests that they might not be the active substances that exert beneficial physiological effects.

## 3. Biological Properties of Flavan-3-Ol Microbial Metabolites

**Figure 2:**
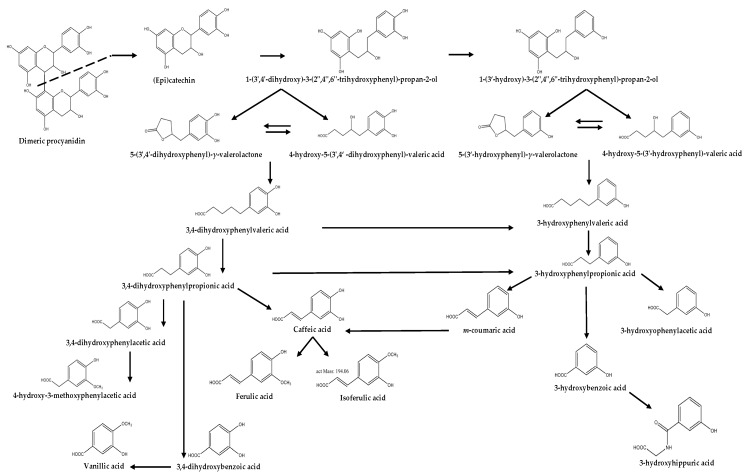
Metabolic pathway of flavan-3-ols in the colon.

After the unabsorbed flavan-3-ols reach the colon, free monomeric forms are released and then become available for microbial transformation (Figure 1). These free monomers are converted by specific bacteria into diphenylpropan-2-ols before being further transformed to phenyl-γ-valerolactones, which are intermediate metabolites that are exclusive to flavan-3-ols (Figure 2). Further reactions, which include breaking the valerolactone ring and dihydroxylation, form various forms of phenylvaleric acids [3,4].

The further bacterial metabolism of phenyl-γ-valerolactones and phenylvaleric acids produces different hydroxylated forms of phenyl and benzoic acids by the loss of carbon atoms from the side chain through ß-oxidation (Figure 2) [3]. The 3,4-dihydroxylated phenolic acids are dehydroxylated at C-4’ and C-3’, producing 3- and 4-monohydroxylated phenolic acids [3]. Metabolites, like vanillic acid, homovanillic acid, hippuric acid, or *p*-coumaric acid, have also been related to the intestinal catabolism of flavan-3-ols (Figure 2) [3]. These low molecular weight phenolic acids are, in contrast to phenyl-γ-valerolactones, not specific to flavan-3-ols, since they can also be formed after the metabolism of other flavonoids [11,12].

After absorption, colonic metabolites undergo phase II metabolism in the liver and their conjugated derivatives reach the organs and tissues, where they will exert their beneficial effects. They can also be eliminated in the urine and faeces [3].

Unlike the native monomeric and, especially, the oligomeric and polymeric flavan-3-ols, colonic metabolites are highly bioavailable in the human organism. In fact, their absorption has been shown to occur at a much higher rate than in the case of the parent compounds [10,13,14,15]. Phenyl-γ-valerolactones and phenylvaleric acids have been previously found at concentrations lower than 1 µM in plasma [15,16,17,18,19]. The smaller phenolics have been mainly found at concentrations lower than 0.5 µmol/L [16,17,18,19], but in some cases, such as phenylacetic acid, protocatechuic acid, and hippuric acid, the concentrations could reach ≈ 40 µmol/L [16,20].

### 3.1. Phenyl-γ-valerolactones and Phenylvaleric Acids

Table 1 presents an overview of the studies that were performed over the last few years regarding the biological activities, and including the physiological importance, of phenyl-γ-valerolactones and phenylvaleric acids that, according to the available literature, are related to the human colonic metabolism of monomeric flavan-3-ols and proanthocyanidins [3,5].

#### 3.1.1. Anti-adhesive Activity

Urinary tract infections are often caused by *Escherichia coli* [29] and cranberry has been commonly used for its treatment, because proanthocyanidins are believed to prevent bacterial adhesion to uroepithelial cells [30,31]. However, since these complex structures are rarely found in high amounts in plasma or urine after ingestion, Mena et al. investigated the anti-adhesive effect of one of its metabolites, 5-(3’,4’-dihydroxyphenyl)-γ-valerolactone, and its conjugated forms [21]. At a concentration of 100 µM, the metabolites significantly inhibited the adherence of uropathogenic *E. coli* to T24 bladder epithelial cells [21]. The most effective derivative at this concentration was 5-phenyl-γ-valerolactone-3’,4’-di-*O*-sulphate, which led to a 30.3 ± 3.6% inhibition of adherence. The unconjugated form exerted the lowest inhibitory effect, with 19.4 ± 10.3% adherence inhibition [21]. At 50 µM, only 5-(3’-hydroxyphenyl)-γ-valerolactone-4’-*O*-sulphate significantly inhibited the adhesion [21].

#### 3.1.2. Anti-inflammatory Activity

Uhlenhut et al. studied the effect of 5-(3’,4’-dihydroxyphenyl)-γ-valerolactone on nitric oxide (NO) production and inducible nitric oxide synthase (iNOS) expression by RAW 264.7 murine macrophages after lipopolysaccharide (LPS) stimulation [22]. NO production and iNOS expression were both inhibited by the microbial metabolite in a concentration-dependent manner (IC_50_ = 1.3 µg/mL, IC_50_ = 3.8 µg/mL, respectively) [22]. Interestingly, the authors also found that the metabolite had high binding capacity to macrophages, endothelial cells, and monocytes [22].

#### 3.1.3. Cardiovascular Protective Effect

Phenyl-γ-valerolactones and phenylvaleric acids could contribute to the preventive effects that were attributed to flavan-3-ols on cardiovascular diseases through hypotensive properties and by attenuating the monocyte adhesion to endothelial cells that are involved in the development of atherosclerosis.

Particularly, two metabolites of (−)-epigallocatechin gallate produced by intestinal microbiota, namely 5-(3,4,5-trihydroxyphenyl)-γ-valerolactone and 5-(3,5-dihydroxyphenyl)-γ-valerolactone, were reported to have hypotensive effects in vivo in rats [23]. The first metabolite that was administered at a concentration of 150 mg/kg significantly decreased systolic blood pressure 2 h after intake when compared to the control group [23]. In contrast, the second metabolite required a higher oral dosage of 200 mg/kg to exert an effect on systolic blood pressure 4 h after its administration [23]. Moreover, hydroxyphenyl valeric acids that were also derived from (−)-epigallocatechin gallate metabolism (5-(3,4,5-trihydroxyphenyl)valeric acid, 5-(3,5-dihydroxyphenyl)valeric acid and 5-(3-hydroxyphenyl)valeric acid) showed strong inhibitory activity of angiotensin I-converting enzyme (ACE) with an IC_50_ higher than that of (−)-epicatechin, but lower than (−)-epigallocatechin gallate [23].

Recently, Lee et al. reported that monocyte-endothelial cell adhesion was dose-dependently prevented after treatment with 5-(3’,4’-dihydroxyphenyl)-γ-valerolactone at concentrations under 30 µM [24]. Two of the main molecules that were involved in the monocyte-endothelial adhesion are vascular cell adhesion molecule (VCAM)-1 and monocyte chemoattractant protein (MCP)-1, and their tumor necrosis factor (TNF-α)-stimulated expression was downregulated after treatments with the phenyl-γ-valerolactone at concentrations between 7.5 and 30 µM [24]. This was possibly due to the parallel downregulation of the phosphorylation of two nuclear factor kappa B (NF-κB) activation signalling regulators as well as NF-κB transcriptional activation [24].

#### 3.1.4. Chemopreventive Effect

Hara-Terawaki et al. investigated the effect of flavan-3-ol microbial metabolites on the proliferation of human cervical cells [25]. Among all the metabolites produced from (−)-epigallocatechin, (−)-epigallocatechin gallate and (−)-epicatechin, 4-hydroxy-5-(3,4,5-trihydroxyphenyl)valeric acid, 5-(3,4,5-trihydroxyphenyl)valeric acid, and 5-(3,4-dihydroxyphenyl)valeric acid showed inhibitory activity on cell proliferation, relative to a negative control set at 100 (DMSO) of 71.9%, 13.5%, and 53.9%, respectively, at 50 µg/mL [25]. Therefore, the authors suggested that the presence of three hydroxyl groups and the aliphatic side chain could be involved in the anti-proliferative effect of the microbial metabolites [25].

A phenyl-γ-valerolactone, 5-(3’,4’,5’-trihydroxyphenyl)-γ-valerolactone, showed a modest anti-proliferative effect in androgen-dependent human prostate cancer cells (LNCaP), with IC_50_ = 117 μM [26]. Moreover, the dihydrotestosterone-induced nuclear translocation of androgen receptor, known to play a key role on prostate cancer development and progression, was suppressed after 100 µM treatment with the microbial metabolite [26].

#### 3.1.5. Immunostimulatory Activity

Some of the positive effects of the intermediate microbial metabolites from flavan-3-ols seem to be as well mediated by direct stimulation of the immune system. In this regard, Kim et al. reported that 5-(3’,5’-dihydroxyphenyl)-γ-valerolactone plays an important role on immunostimulation, because the splenic NK cell cytotoxic activity against murine lymphoma YAC-1 cells was significantly increased after its oral administration (10 mg/kg) in comparison to the control and (−)-epigallocatechin treatment groups [27]. In addition, 5-(3’,5’-dihydroxyphenyl)-γ-valerolactone upregulated the splenic production of IFN-γ, a T cell growth factor and effector of CD4⁺ T cells [27]. CD4⁺ T cell activity also increased after incubation with 10 µM 5-(3’,5’-dihydroxyphenyl)-γ-valerolactone for 72 h, as well as it did with other microbial metabolites lacking the 4’-hydroxyphenyl group on the B ring [27].

#### 3.1.6. Neuroprotective Effect

The in vitro stimulation of nerve cell proliferation and differentiation by phenyl-γ-valerolactones was recently reported by Unno et al. [28]. Particularly, only 0.05 µM 5-(3’,5’-dihydroxyphenyl)-γ-valerolactone could enhance human SH-SY5Y neuroblastoma cell number, but at concentrations that were higher or equal to 1.0 µM, the effect was reduced [28]. The same concentration of the microbial metabolite, including its sulfated form, increased the length and number of neurites in SH-SY5Y as compared to control cells, whereas the glucuronide form could only increase the number of neurites [28].

### 3.2. Phenolic Acids

Table 2 presents the biological activities reported for smaller phenolic acids result of microbial catabolism of flavan-3-ols.

#### 3.2.1. Anti-adhesive Activity

The inhibitory activity against the adherence of uropathogenic bacteria *E. coli* to uroepithelial cells has also been tested with low molecular weight phenolics. In particular, catechol, benzoic acid, vanillic acid, phenylacetic acid, and 3,4-dihydroxyphenylacetic acid (3,4-DHPA) inhibited *E. coli* adherence in a concentration-dependent manner from 100 to 500 µM [32]. The inhibitory effect appeared to be more pronounced in the case of gallic acid, phenylacetic acid, and 3,4-DHPA, which induced up to 40.6 ± 20.2%, 40.6 ± 10.1%, and 37.0 ± 20.5% adherence inhibition at 500 µM, respectively [32].

#### 3.2.2. Antidiabetic Effect

The antidiabetic effects of various microbial metabolites from flavan-3-ols have been investigated from different perspectives, and the involved mechanisms are apparently not only related to their antioxidant properties, but also to their ability to modulate different signalling pathways.

Scazzocchio et al. assessed the role of protocatechuic acid in the glucose transport in adipocytes [33]. Incubation of the metabolite at 100 µM for 18 h with human and murine adipocytes treated with oxidized LDL (oxLDL) showed that protocatechuic acid significantly reversed the detrimental effects of oxLDL on glucose uptake and glucose transporter type 4 (GLUT4) translocation. In addition, when both adipocytes were insulin-stimulated and also when human adipocytes were not insulin-stimulated, protocatechuric acid significantly removed the detrimental effect of oxLDL on adiponectin secretion [33]. Glucose uptake increased in a significant and concentration-dependent manner in non-oxLDL-treated human and murine adipocytes in the absence of insulin up to 40% and 60%, respectively [33]. These results suggested an insuline-like activity. The reduced peroxisome proliferator-activated receptor-γ (PPARγ) mRNA expression and activity induced by oxLDL was also counteracted, and its inhibition blocked both the adiponectin and GLUT4 upregulation, which suggests a direct involvement on the insuline-like activity of protocatechuic acid [33].

The effect of low molecular weight phenolics on beta cell functionality has also been assessed. Fernández-Millán et al. reported a significant increase in glucose-induced insulin secretion in INS-1E pancreatic beta cells and isolated rat islets after treatment with 3,4-DHPA and 3-hydroxyphenylpropionic acid (3-HPP) at low concentrations (5 and 1 µM, respectively) [34]. Moreover, in the presence of oxidative stress that was induced by t-BOOH, both 3,4-DHPA and 3-HPP restored glucose-stimulated insulin secretion to control levels and significantly reduced t-BOOH-induced cell death as well as ROS and carbonyl group production [34]. Protein kinases, specifically protein kinase C (PKC) and extracellular signal–regulated kinases (ERK), could be involved, since their phosphorylation levels were enhanced after treatment and their pharmacological inhibition blocked the increased insulin secretion that was induced by the metabolites [34].

Carrasco-Pozo et al. evaluated the protective effect of 3,4-DHPA on Min6 pancreatic beta cells dysfunction that was induced by high cholesterol, and the metabolite at 250 µM could indeed prevent the decrease in insulin secretion induced by high cholesterol [35]. Furthermore, it prevented the cholesterol-induced cytotoxicity and apoptosis in a concentration-dependent manner, and also prevent oxidative stress and mitochondrial dysfunction [35]. Nuclear factor (erythroid-derived 2)-like 2 (Nrf2) translocation to the nucleus was increased by around six-fold relative to control, which suggests that it contributes to the antioxidant protection exerted by 3,4-DHPA [35].

Recently, Bitner et al. reported that hippuric acid and homovanillic acid, as well as the phenylvaleric acid 5-phenylvaleric acid, more effectively enhanced glucose-stimulated insulin secretion (GSIS) in beta cells than (−)-epicatechin at concentrations between 5 and 100 µM (hippuric acid between 5 and 50 µM) [38]. Contrary to (−)-epicatechin, the microbial metabolites enhanced GSIS without enhancing beta cell mitochondrial respiration or increasing expression of mitochondrial electron transport chain components [38]. In addition to the stimulation of beta cell function, the microbial metabolites stimulated glucose utilization in skeletal muscle and they preserved mitochondrial function after insulation [38].

Álvarez Cilleros et al. investigated how flavan-3-ol-derived low molecular weight phenolics influenced the mechanisms related to the glucose homeostasis in rat renal NRK-52E cells since kidneys are involved in the maintenance of glucose homeostasis [36]. The authors found out that 2,3-dihydroxybenzoic acid (2,3-DHB) at 20 µM reduced cellular glucose uptake in a similar way to the sodium-glucose co-transporter-2 (SGLT-2) antagonist phlorizin, without altering the expression levels of SGLT-2 and glucose transporter-2 (GLUT-2) [36]. Glucose production was significantly reduced and the levels of the gluconeogenic enzyme phosphoenolpyruvate carboxykinase (PEPCK) were also reduced [36]. In addition to the effects on glucose uptake and production, the effect of the microbial metabolite on the activation of proteins from the insulin signalling pathway was evaluated. 2,3-DHB significantly increased insulin receptor (IR), insulin receptor substrate 1 (IRS-1) tyrosine phosphorylated, and total protein levels, as well as the phosphorylated levels of protein kinase B (Akt) and glycogen synthase kinase-3 (GSK3), and subsequently reduced the glycogen synthase (GS) phosphorylated levels [36]. Moreover, the authors showed that Akt was involved in the modulation by 2,3-DHB of both PEPCK levels and glucose production in the NRK-52E cells [36].

Similarly, in another study from Álvarez-Cilleros et al., when renal tubular NRK-52E cells were treated with high glucose levels, 3,4-DHPA at 10 µM restored the induced alteration in glucose uptake and production and increased tyrosine phosphorylated and total levels of IR [37]. Moreover, it restrained the inhibition of the phosphatidylinositol-3-kinase (PI3K)/Akt pathway that was involved in the insulin signalling cascade and counteracted the high glucose-induced downregulation on 5′-AMP-activated protein kinase (AMPK) phosphorylation and upregulation on PEPCK [37]. When Akt and AMPK were inhibited the protective effect of 3,4-DHPA was abrogated, which suggests that they play a key role on the preservation of renal tubular functionality, the modulation of the glucose homeostasis by the metabolites, together with the attenuation of the insulin signalling blockade [37].

#### 3.2.3. Antiglycative Activity

Advanced glycation end-products (AGEs) contribute to the occurrence of several pathologies, such as diabetes and rheumatoid arthritis [83]. For this reason, the effect of phenolics against their formation has been evaluated. Dihydroferulic acid has been shown to significantly inhibit albumin glycation at a concentration of 10 µmol/L, and the combination of 3-hydroxyphenylacetic acid (3-HPA), 3,4-DHPA, and homovanillic acid at 2.0 µmol/L has also shown this [39]. 3,4-DHPA at 1 mM exerted an inhibitory activity against AGEs that was significantly lower than that of rutin and quercetin, but significantly higher than the positive control aminoquanidine, 3-HPA and homovanillic acid [40].

#### 3.2.4. Anti-inflammatory Activity

Prevention of inflammation by microbial low molecular weight phenolics has been shown to occur by modulating the mediators of inflammation and additionally the signal transduction pathways involved.

Yang et al. reported that *trans*-caffeic acid and 3,4-dihydroxyphenylpropionic acid (3,4-DHPP) inhibited the NO production in LPS-activated RAW264.7 cells (IC_50_ = 224.85–689.91 µM) [41]. In the same cellular model and in dendritic D2SC/I cells, Ho et al. reported the same anti-inflammatory effects for *trans*-caffeic, and for the metabolites 3,4-DHPA, protocatechuic acid, and *p*-coumaric acid [46].

The secretion of another inflammatory mediator, TNF-α, induced by LPS in THP-1 monocytes, was reduced by benzoic acid-sulfate, vanillic acid-glucuronide, and protocatechuic acid-3-sulfate in a study from di Gesso et al. [42]. Moreover, four different combinations of metabolites that included 4-hydroxybenzoic acid or protocatechuic acid or both could also significantly reduce TNF-α secretion, but, interestingly, to a greater extent than the metabolites alone, which showed no significant effect [42].

Wang et al. showed that protocatechuic acid at 5, 10, and 20 µM dose-dependently inhibited the production of the inflammatory mediators TNF-α, interleukin 6 (IL-6), interleukin 1 beta (IL-1ß), and prostaglandin E_2_ (PGE_2_) in LPS-stimulated BV2 microglia [43]. The LPS-induced expression of toll-like receptor 4 (TLR4), activation of NF-κB, and mitogen-activated protein kinases (MAPKs) in BV2 microglial cells was furthermore suppressed by protocatechuic acid, which suggests that these signaling pathways are actively involved in its anti-inflammatory activity [43]. The same metabolite at 5, 10, and 20 µM was also shown to inhibit LPS-induced production of IL-6 and interleukin 8 (IL-8) in human gingival fibroblasts, secondarily to its ability to activate PPAR-γ [44].

In a 2,4,6-trinitrobenzenesulfonic acid (TNBS) model of rodent inflammatory bowel disease, protocatechuic acid administered at 30 and 60 mg/kg improved the TNBS-induced colitis and reduced oxidative stress that was measured as GSSG/GSH ratio [45]. The antioxidant enzymes expression increased after treatment with protocatechuic acid when compared to the TNBS group and additionally the transcription factor Nrf2 protein levels [45]. The TNBS-induced increase in colonic expression of the inflammatory markers IL-6, TNF-α, IL-1β, and cyclooxygenase-2 (COX-2) was reversed [45]. The SphK/S1P signaling pathway seems to play a key role, since TNBS-induced an increase in mRNA levels, an increase in protein concentration and immunohistochemical labelling for SphK1 was prevented by protocatechuic acid [45]. Similarly, the increase in S1P production and expression of S1P receptor 1 and S1P phosphatase 2 were significantly reversed [45]. Related signaling pathways might be also involved, since protocatechuic acid reversed the TNBS-induced increase of phosphorylated signal transducer and the activator of transcription 3 (STAT3), NF-κB p65 subunit expression, and phosphorylation of Akt and ERK [45]. In a similar model, gallic acid that was given at 20–60 mg/kg improved the TNBS-induced colitis in mice and inhibited inflammation reducing proinflammatory cytokines, such as IL-1 and IL-6, and increased the anti-inflammatory cytokines *via* inhibiting the NF-κB pathway [47].

#### 3.2.5. Antioxidant Activity

Low molecular weight phenolics have been investigated for their antioxidant activity in vitro through different assays that are able to measure their scavenging capacity against non-biological radicals, including 2,2-diphenyl-1-picrylhydrazyl radical assay (DPPH• assay), the 2,2-azinobis-(3-ethylbenzothiazoline-6-sulphonate) radical cation assay (ABTS•+ assay), and ferric reducing antioxidant power (FRAP assay), as well as their scavenging capacity against biological oxidants, such as superoxide radicals scavenging (O2·-).

One of the studied microbial metabolites of flavan-3-ols showing high antioxidant activity is 3,4-DHPA. This metabolite was able to scavenge ABTS [48], DPPH [40,49,50], as well as reduce Fe^3+^ ions to Fe^2+^ ions [40,48,50], being effective, even at low concentrations, like 5 µM, and, in some cases, showing an equivalent scavenging activity to flavonoids, like rutin or quercetin [40,49].

Other derivatives of microbial catabolism of flavan-3-ols that have also shown antioxidant activity are caffeic acid [41], 3,4-DHPP [41], gallic acid [48], pyrogallol [48], homovanillic acid [40], protocatechuic acid [49], as well as vanillic and ferulic acids [50]. In some studies, metabolites, such as 3-HPA [40,49], 3-HPP [41], and hippuric acid [49], showed no antioxidant activities.

Although the evaluation of the antioxidant activity of all these compounds has been performed while using different assays, as well as at different concentrations and conditions, it is clear that low molecular weight phenolics can act as antioxidants. As suggested by many authors, the number of hydroxyl groups can be involved in their antioxidant activity.

#### 3.2.6. Anti-proliferative Activity and Cytotoxicity

The anti-proliferative activity of low molecular weight phenolics has been assessed in a variety of cellular models, including murine macrophages as well as human cancer cell lines. For a better classification of the biological activities of metabolites, the studies assessing the anti-proliferative activity on human cancer cell lines are presented in a following section about chemopreventive effects.

In a study from Varì et al., protocatechuic acid at a concentration of 25 µM counteracted the cytostatic and cytotoxic effect of oxLDL in J774A.1 macrophages [51]. The oxLDL induced an increased apoptosis as well as oxidative stress, and both were reduced after treatment with the metabolite, although the ROS production could only be abolished during the first six hours of treatment [51]. The metabolite at 25 µM could reduce the oxLDL-induced activation of p53 protein and counteract the overexpression of the main p53 target genes: p66Shc and Bax [51]. The activation of the c-Jun N-terminal kinases (JNK)/Nrf2 pathway could play a key role in the anti-apoptotic effects of protocatechuic acid, since its inhibition caused a change in the expression of phosphorylated p53, Bax and the active form of caspase-3 comparable to levels that were found in cells only treated with oxLDL [51]. This inhibition also suppressed the effect of protocatechuic acid on the reduction of oxLDL-induced ROS levels [51].

Liu et al. also recently reported the anti-proliferative effects of protocatechuic acid in preventing or treating asthma airway remodeling, since it suppressed the proliferation of airway smooth muscle cells and extracellular matrix protein deposition in transforming growth factor-beta1 (TGF-ß1)-mediated airway smooth muscle cells via the inactivation of Smad2/3 signaling pathway [52].

#### 3.2.7. Cardiovascular Protective Effect

Low molecular weight phenolics could have a positive effect on the prevention of cardiovascular disease through protection of the heart and the endothelial function, through antithrombotic effects, as well as through their previously presented anti-inflammatory activity.

On the one hand, the anti-platelet and antithrombotic potential of protocatechuic acid were confirmed in a study from Kim et al. [53]. This metabolite at concentrations between 5 and 25 µM significantly decreased stress-induced platelet aggregation in isolated human platelets, which was mediated by blocking the interaction between von Willebrand factor (vWF) and platelet receptor glycoprotein Ib [53]. The increase of intracellular calcium induced by shear stress was dose-dependently attenuated by protocatechuic acid. Moreover, this metabolite inhibited shear-induced granular secretion from dense and α-granules and attenuated GP IIb/IIIa activation. Interestingly, protocatechuic acid did not inhibit platelet aggregation induced by other endogenous agonists [53]. In an in vivo rat arterial thrombosis model, the antithrombotic effects of PCA were confirmed and this metabolite did not show increased risk of bleeding [53].

Similarly, 4-methylcatechol has been reported to also have anti-platelet effects [60]. When testing its effect on whole blood platelet aggregation induced by arachidonic acid, this metabolite was much more active than acetylsalicylic acid (IC_50_ = 3 µM vs. IC_50_ = 25 µM, respectively) [60]. This could be confirmed by the authors in an *ex ovo* thrombotic model that mimics the in vivo situation [60]. Moreover, 4-methylcatechol was shown to interfere with calcium intracellular trafficking, and this seemed to be its main mechanism of action. As the authors suggest, the methyl group probably does not play any role in the anti-platelet potential, but the hydroxyl groups in positions 1 and 2 seem to be important [60].

On the other hand, the cardioprotective effects have been attributed to protocatechuic acid by Semaming et al. [54]. When administered to type 1 diabetic rats at a dosage of 50 and 100 mg/kg, it significantly increased the fractional shortening and the left ventricular ejection fraction when compared to non-treated diabetic rats after 8 and 12 weeks, and the effects were partially comparable to those after treatment with insulin [54]. Moreover, the low-frequency:high-frequency ratio was significantly decreased when compared with the non-treated diabetic rats. The authors also found a significant decrease of plasma HbA1c and cardiac MDA levels, an improvement of cardiac mitochondrial function, and an increase in anti-apoptotic BCL2 expression [54].

In another study, the same metabolite was shown to have a positive effect on diabetic cardiomyopathy in type 2 diabetic rats when orally administered (50 and 100 mg/kg) [56]. Protocatechuic acid exerted hypoglycemic and insulin-sensitizing activities *via* the stimulation of IRS1/PI3K/AKT/AMPK/GLUT4/P 38 signaling pathway in the skeletal muscle and anti-inflammatory effects mediated by the downregulation of the poly (ADP-ribose) polymerase (PARP)/PKC/NF-κB signaling cascade in the myocardial tissue [56]. Moreover, antioxidant and radical scavenging effects in the myocardial tissue of type 2 diabetic rats were also attributed to the administration of protocatechuic acid [56].

The vasodilatory effect of microbial metabolites has been well assessed. Najmanová et al. performed an in vitro study where 3-HPP at 100 nM exerted the strongest vasodilatory activity on isolated aortic rings from all tested metabolites, and both endothelium and NO were found to play a role [55]. In a study from Pourová et al., 3,4-DHPA and 4-methylcatechol were reported to induce vasorelaxation in pre-contracted aortic rings and mesenteric artery [59]. The metabolites exerted similar effects in both aortic rings (96.5% and 96.8% relaxation, respectively) and the mesenteric artery (101.4% and 98.3% relaxation, respectively) [59]. The half maximal effective concentrations of 3,4-DHPA to induce vasorelaxation in aortic rings and mesenteric artery were EC_50_ = 22.4 ± 1.3 µM and EC_50_ = 34.2 ± 5.6 µM, respectively [59]. For 4-methylcatechol, these were EC_50_ = 49.1 ± 3.3 µM and EC_50_ = 30.5 ± 1.4 µM, respectively [59]. Unlike 4-methylcatechol, the vasorelaxant effects of 3,4-DHPA were partially dependent on endothelium and reduced after atropine administration [59].

In vivo models in rats show a decrease of arterial blood pressure in both normotensive and spontaneously hypertensive rats after bolus (2.5–25 mg/kg) and 5-min. infusion (5 mg/kg/50 µL/min., mimicking gastrointestinal absorption) administration of 3-HPP [55]. Similarly, the administration of 3,4-DHPA and 4-methylcatechol as a bolus or as an infusion dose-dependently decreased blood pressure in rats [59].

3-HPP was also reported to be beneficial for endothelial function by Qian et al. [57]. Particularly, 1 µM 3-HPP was able to maintain the NO production stimulated by insulin in human aortic endothelial cells under glucotoxic conditions, and additionally to increase endothelial nitric oxide synthase (eNOS) and Akt phosphorylation [57].

Álvarez Cilleros et al. also reported an increased NO production too, but, in this case after the treatment of EA.hy927 human endothelial cells with 10 µM 3,4-DHPA or with a mix of that metabolite with 2,3-dihydroxybenzoic acid (2,3-DHB) and 3-HPP (12 µM) [58]. AMPK and Akt seem to be key mediators, since their inhibition blocked the NO production as well as eNOS phosphorylation [58]. Under oxidative stress that is induced by t-BOOH, both 3,4-DHPA and the mix of the metabolites reversed the induced endothelial dysfunction by preventing increased ROS generation and the activation of signalling pathways related to oxidative stress [58].

#### 3.2.8. Chemopreventive Effect

The potential cancer preventing effect of low molecular weight phenolics has often been studied in in vitro models of intestinal cell lines. Henning et al. found that the proliferation of HCT116 colon cancer cells was inhibited by 3,4-DHPA and 3-O-methylgallic acid [61]. The concentrations of 3,4-DHPA that were needed for a significant inhibition of cell proliferation were 200 and 300 µmol/L, but the concentration exhibiting 50% inhibition was higher than 400 µmol/L. However, 3-O-methylgallic acid, had a IC_50_ = 260 µmol/L [61]. When 3,4-DHPA was combined with epigallocatechin, the inhibitory effects significantly increased when compared to the individual treatments [61].

Rosa et al. also reported 3,4-DHPA to induce the largest reduction of cell viability of HT-29 human colon adenocarcinoma cells at concentrations between 2.5 and 100 µM (up to 66% reduction) [50]. *p*-Coumaric acid, vanillic acid, and ferulic acid could also reduce cell viability [50]. The metabolites modulated the cell cycle, since 3,4-DHPA, *p*-coumaric acid, vanillic acid (10 and 100 µM), and additionally ferulic acid (10 µM) decreased the cell number in S phase [50]. Relative apoptosis rate data showed that *p*-coumaric acid and ferulic acid at 10.0 μM promoted increased apoptosis [50]. In a study from López de las Hazas et al., late-stage apoptosis of HT-29 colon cancer cells was reported to be also induced by 4-hydroxyphenylacetic acid [62].

The anti-proliferative activity, cell cycle arrest and apoptosis of caffeic acid, 3-phenylpropionic acid (3-PP), and benzoic acid in Caco-2 human colon cancer cells were tested by Sadeghi Ekbatan et al. [64]. Only caffeic acid reduced cell proliferation by 50% (EC_50_ = 460 ± 21.88 µM), while 3-PP and benzoic acid reduced it at a concentration of 1000 μM [64]. Cell-cycle arrest was induced at the S-phase by caffeic acid at 100 μM and by 3-PP at 500 μM [64]. Regarding apoptosis, caffeic acid at 1000 µM activated caspase-3, and the mitochondrial DNA content was reduced by 3-PP at 1000 µM [64].

Since angiogenesis, the process by which new blood vessels are formed is involved in the progression of tumors, Hu et al. evaluated the anti-angiogenic activity of protocatechuic acid in vitro as well as in an in vivo animal model [63]. In the in vitro model with human umbilical vein endothelial cells (HUVEC), protocatechuic acid inhibited the vascular endothelial growth factor (VEGF)-induced proliferation, the formation of capillary tubes, and the migration and invasion of HUVECs [63]. The mechanisms that are involved might be related to its ability to attenuate ROS production (6.25–100 μM) as well as by interfering with VEGF-dependent Akt/MMP2 and ERK pathways [63]. The in vivo model in zebrafish showed that, at 25 μM, protocatechuic acid also had a significant anti-angiogenic effect when compared with the control group, possibly through downregulation of the angiogenesis-related signal transduction pathways of VEGFα-VEGF Receptor 2 and angiotensin 2-Tie2 receptor [63].

Protocatechuic acid at up to 30 µM was furthermore able to reduce cell viability of ovarian cancer cells OVCAR-3 in a dose-dependent manner (IC_50_ = 10.7 µM) and it induced apoptosis [65]. Furthermore, it induced the cell cycle arrest in G_2_/M phase [65]. The increased apoptosis was accompanied by the activation of PARP and caspase-3, the upregulation of Bax and downregulation of B-cell lymphoma 2 (Bcl-2) in OVCAR-3 cells [65]. Additionally, autophagy-related protein LC3-II was upregulated and the intracellular ROS levels decreased, while those of glutathione increased [65].

#### 3.2.9. Modulation of Drug Metabolizing Enzymes

The correct functioning of drug metabolism is essential in transforming and removing foreign substances from the organism. The expression and functions of the enzymes taking part in this process can be altered in some physiological situations [84], and the enhancement of their capacity could protect against pathologies, such as cancer [85]. Therefore, the effect of phenolic compounds on drug metabolizing enzymes has been previously evaluated.

In a study from Miene et al., the expression of two drug metabolizing enzymes, namely glutathione S-transferase theta-2 (GSTT2) and COX-2, in LT97 human colon cells was modulated by 3,4-DHPA and 3,4-DHPP [66]. Microbial metabolites both increased the GSTT2 mRNA expression up to 1.7-fold (2.5 and 5 µM). On the contrary, COX-2 mRNA was reduced after the incubation with both microbial metabolites [66]. COX-2 protein expression was dose-dependently reduced after treatment with 3,4-DHPA (5–10 µM) and 3,4-DHPP (15–25 µM) during 48 h, but no effect was seen after 24 h incubation [66].

3,4-DHPA also modulated the gene expression of drug metabolizing enzymes as well in Hepa1c1c7 cells in a study from Tang et al. [49]. The mRNA levels of glutamate-cysteine ligase catalytic subunit (GCLC), heme oxygenase 1 (HO-1), and NADH: quinone oxidoreductase-1 (NQO1) significantly increased at concentrations of the metabolite of 5, 10, and 25 µM, respectively [49]. The mRNA levels of cystine/glutamate anti-porter (xCT) and Cytochrome P450 1A1 (CYP1A1) were additionally increased, but at higher concentrations of 100 and 250 µM, respectively [49]. The GSH-activity in RL34 normal hepatocytes was also increased in a dose-dependent manner, and reached an activity of 1.4-fold higher than control levels [49]. Moreover, 3,4-DHPA reduced the cytotoxic effect of peroxide in Hepa1c1c7 cells in a concentration-dependent manner, and cell viability was completely restored to control levels at a concentration of 100 µM [49].

Furthermore, Xue et al. reported that 3,4-DHPA was able to inhibit oxidative stress in acetaminophen-induced hepatotoxicity in rats by decreasing malondialdehyde levels and by dose-dependently increasing the antioxidant enzymes glutathione (GSH), glutathione peroxidase (GPx), glutathione *S*-transferase (GST), and superoxide dismutase (SOD) to prevent the decrease of UDP-glucuronosyltransferase (UGT) and sulfotransferase (SULT)’s activity induced by acetaminophen [67].

The effect of 3,4-DHPA on aldehyde dehydrogenase was also investigated by Liu et al., and the authors found that, after treatment of mouse hepatoma Hepa1c1c7 cells, the total activity of ALDH increased, as well as the expression of ADLH1A1, ADLH2 and ALDH3A1 [68]. The authors also showed that 3,4-DHPA activates the signalling pathways of Nrf2 and AhR to possibly increase the expression of the ALDH genes, but inhibits that of NF-κB [68].

In addition to 3,4-DHPA, protocatechuic acid was also shown to modulate drug metabolizing enzymes. Ibitoye et al. administered protocatechuic acid to rats at 10 and 20 mg/kg and it significantly reversed menadione-induced increases in superoxide ions and hydrogen peroxide in addition to decreases in the activities of SOD and catalase (CAT) [69]. The metabolite reversed the induced decrease in GST and NQO-1, and significantly increased Nrf-2, Akt, and PI3K, which suggests that this is a possible pathway for the increasing antioxidant and phase II metabolizing activities [69].

#### 3.2.10. Modulation of Intestinal Microbiota

Ma et al. investigated the effect of one microbial metabolite, ferulic acid, on faecal microbiota of HFD-induced obese mice [70]. The authors reported that FA significantly reduced the ratio of Firmicutes to Bacteroidetes when compared to the obese control group [70].

The growth of two probiotic (*Lactobacillus rhamnosus* and *L. acidophilus)* and two pathogenic bacteria (*Escherichia coli* and *Salmonella enterica* serovar Typhimurium) in the presence of gallic acid, vanillic acid, ferulic acid, and protocatechuic acid was evaluated in another study [71]. The results showed that the minimal inhibitory concentration (MIC) and minimal bactericidal concentration (MBC) of phenolic acids against *E. coli* and *S*. Typhimurium showed similar values of 15-30 mmol/L [71]. However, the MICs were higher for probiotics (mostly >35 mmol/L), except in the case of ferulic acid (MIC = 20 mmol/L) [71]. The MBC were, in this case, higher than 35 mmol/L for all metabolites. Furthermore, the presence of the metabolites in MRS broth without dextrose promoted the growth of lactobacilli [71]. Therefore, the compounds selectively inhibited the growth of pathogenic bacteria without affecting the viability of probiotics [71].

#### 3.2.11. Modulation of Lipid Metabolism

The effect of ferulic acid on lipid metabolism in high-fat diet-induced obese mice was studied by Naowaboot et al. [72]. The results show that this metabolite at a dosage of 25 and 50 mg/kg reduced the serum lipid level (total cholesterol, triglycerides, and non-esterified fatty acids), as well as liver cholesterol and triglyceride when compared to the obese mice control group [72]. This could be explained by the modulation of the lipogenic genes: the expression of sterol regulatory element-binding protein 1c (SREBP1c), fatty acid synthase (FAS), and acetyl-CoA carboxylase (ACC) were reduced by the metabolite [72]. Moreover, hepatic carnitine palmitoyltransferase 1a (CPT1a) gene and peroxisome proliferator-activated receptor alpha (PPARα) proteins were up-regulated, being involved in the stimulation of ß-oxidation genes [72].

Similarly, after a 12-week treatment of high-fat diet-induced obese mice with 30 mg/kg of ferulic acid, serum total cholesterol, triglycerides, and LDL cholesterol significantly decreased when compared to obese control group [70]. Total cholesterol and triglyceride levels in liver were as well significantly reduced after treatment with the metabolite [70]. The mechanism could be due to the significant increase of aryl hydrocarbon receptor (AHR)’s mRNA expression, which is thought to regulate lipid metabolism and inhibit FAS and SREBP1c. Indeed, after treatment with ferulic acid the mRNA expression of FAS and SREBP-1c decreased [70].

#### 3.2.12. Neuroprotective Effect

Through their antioxidant, anti-inflammatory and anti-apoptotic properties, low molecular phenolics could also exert a protective effect against neuronal diseases. In a study from Verzelloni et al., the prevention of cytotoxicity induced by 2,3-dimethoxy-1,4-naphtoquinone (DMNQ) was tested on human neuroblastoma SK-N-MC cells after treatment with pyrogallol, 3,4-DHPP, dihydroferulic acid, 3-hydroxyphenylacetic acid (3-HPA), 3,4-DHPA or homovanillic acid [39]. Although all of the metabolites had a positive impact on the survival of human neuroblastoma cells, only 3-HPA did it in a concentration-dependent manner. Dihydroferulic acid at 20 µmol/L induced the greatest increase in cell survival (17.07 ± 4.2% at 20 µmol/L), followed by pyrogallol (12.0 ± 2.5% at 20 µmol/L) and 3,4-DHPA (11.0 ± 0.6 and 11.0 ± 2.2% at 10 and 20 µmol/L, respectively). Different combinations of metabolites at a final concentration of 1.5 µmol/L also exerted significant protection on neuroblastoma cells [39].

In a model of neuroinflammation, SH-SY5Y human neuroblastoma cells were exposed to SIN-1-induced nitrosative stress and treated with 3,4-DHPA, 3-HPP, and 3-HPA [74]. All metabolites at concentrations between 0.1 - 10 µM enhanced cell viability when compared to control group, but 3,4-DHPA had the strongest effect at 10 µM not only after short time exposure, but also after more prolonged time exposure to SIN-1 [74]. The increase in caspase-3 activation induced by SIN-1 was also reduced when the SH-SY5Y cells were treated with 10 µM 3-HPP [74]. Possible mechanisms are the reduction of SIN-1-induced increase of p38 phosphorylation by 3-HPA and of ERK1/2 phosphorylation by all metabolites [74].

The same cell line was exposed to H_2_O_2_ in a study from González-Sarrías et al. and treated with 3,4-DHPP, 3,4-DHPA, and gallic acid, among other polyphenol-derived metabolites [75]. The negative effects of H_2_O_2_ on SH-SY5Y neuroblastoma cell viability were reverted and the percentage of late apoptosis decreased up to approximately 12% with 10 µM pretreatment or cotreatment with the metabolites as compared with H_2_O_2_ treatment alone [75].

In primary cultures of cerebellar granule neurons, 4-hydroxybenzoic acid (100 and 200 µM) and protocatechuic acid (50–200 µM) protected cells from oxidative stress induced by H_2_O_2_ [76]. Under conditions of nitrosative stress typical from inflammative situations in the central nervous system, only protocatechuic acid (10–300 µM) was neuroprotective [76]. However, under conditions of excitotoxicity only 4-hydroxybenzoic acid prevented neuronal death [76]. The ability of these metabolites to alter NO production in a BV2 microglial cell line following treatment with LPS was also tested, and only protocatechuic acid at 100 µM exerted anti-inflammatory activity reducing NO production [76].

In a lysolecithin (LPC)-induced model of inflammation in hippocampal neurons, gallic acid and valeric acid, at 1 and 0.2 µM, respectively, inhibited the LPC-induced demyelination and promoted the formation of myelin, as well as neurite outgrowth [78].

In vivo, the progress of hippocampal neurodegeneration in the brain of diabetic rats was reduced after the administration of 20 and 40 mg/kg gallic acid and *p*-coumaric acid, respectively [73]. These metabolites exerted antioxidant, anti-inflammatory, and anti-apoptotic activities, which probably mediated the protective effects on the hippocampus [73].

Kho et al. recently also evaluated in vivo the effects of protocatechuic acid at a dose of 30 mg/kg on global cerebral ischemia-induced hippocampal neuronal death in rats [77]. The protocatechuic acid-treated rats showed 51%, 58%, 75%, and 76% fewer degenerating neurons than in the vehicle-treated group in the hippocampal areas cornu ammonis 1, subiculum, cornu ammonis 3, and dentate gyrus, respectively [77]. Additionally, ischemia-induced blood-brain barrier disruption was prevented and the induced inflammatory responses mediated by microglia and astrocytes. Oxidative injury was reduced by the metabolite, and the GSH levels after the treatment increased 37% [77].

#### 3.2.13. Osteoprotective Effect

Some low molecular weight phenolics could be effective in preventing bone resorption. In particular, protocatechuic acid was able to inhibit osteoclastogenesis and bone loss in in vivo and in vitro models in a study from Park et al. [79]. At concentrations between 1 and 25 µM, protocatechuic acid dose-dependently inhibited RANKL-induced osteoclast differentiation in mouse bone marrow macrophages and blocked the bone-resorbing activity of mature osteoclasts, probably by suppressing JNK signaling, c-Fos stability, and the expression of osteoclastic marker genes [79]. Mice that were treated with protocatechuic acid efficiently recovered from LPS-induced bone loss in vivo [79].

In another study from Wu et al., protocatechuic acid was also shown to suppress osteoclast differentiation and induce apoptosis in mature osteoclasts [80]. It dose-dependently reduced the Tartrate-resistance acid phosphatase (TRAP) activity induced by RANKL and multinucleated osteoclast formation at a concentration up to 10 µM, as well as ROS and lipid peroxide levels with an increase in antioxidant status [80]. Osteoclast specific markers and transcription factors’ expression was significantly downregulated when compared to that of RANKL, as well as MAPK activation and the expression of inflammatory proteins [80]. Oxidative stress and inflammation were shown to be regulated by protocatechuic acid through regulating the transcription factor Nrf-2 [80]. Moreover, in mature osteoclasts it induced apoptosis by the loss of mitochondrial membrane potential, cytochrome c release, and caspase activation [80].

#### 3.2.14. Renoprotective Effects

Several studies tried to define the preventive properties of microbial phenolic metabolites on diabetic nephropathy, which is a common complication in diabetic patients. In a recent study from Álvarez-Cilleros et al., the renal proximal tubular NRK-52E cells were treated under high glucose levels with 3,4-DHPA in order to assess its protective effect on the redox status [81]. 3,4-DHPA at 10 µM reversed the glucose-induced ROS production and the decrease of antioxidant defenses [81]. SIRT-1 and NADPH-oxidase-4 (NOX-4) appear to play a key role in 3,4-DHPA-mediated renal protection [81].

Protocatechuic acid could also have beneficial effects on renal disease, as shown by Ma et al. [82]. At concentrations between 5 and 10 µM, it dose-dependently inhibited the high glucose-induced proliferation of human mesangial cells and suppressed extracellular matrix expression (type IV collagen, laminin, and fibronectin) [82]. Similarly, the levels of ROS and malondialdehyde induced by high glucose were decreased [82]. The mechanistic proposed by the authors is related to the p38 mitogen-activated protein kinase (MAPK) signaling pathway, since protocatechuic acid downregulated their phosphorylation levels in high glucose-stimulated mesangial cells [82].

## 4. Predictive Potential

In addition to the fact that they are highly bioavailable, when studying the biological activities of flavan-3-ol microbial catabolites, it is important to consider their physiological concentration in plasma after their absorption. Many of the bioactivity studies performed used metabolite concentrations that were much higher than the ones reported in plasma [15,16,17,18,19,20], therefore making the results difficult to relate to health effects in humans.

However, it is worth mentioning that microbial low molecular weight phenolics, in contrast to phenyl-γ-valerolactones, can be formed not only from flavan-3-ols, but also after colonic degradation of many other polyphenols present in food. Therefore, they can be formed from different sources and absorbed in the colon in a continuous manner, which could suggest a higher plasma concentration in the actual physiological conditions.

Interestingly, Uhlenhut and Högger reported a high binding capacity of a phenyl-γ-valerolactone to macrophages, monocytes, and endothelial cell, which made the authors suggest this intracellular accumulation to be the reason why in vivo bioactivity appears at plasma concentrations that exert no effect in vitro [22].

Some authors assessed the effects of mixtures of metabolites [26,39,42,58,61,62,64,71], and reported that some of these combinations had greater activities than the individual compounds. This is also important to consider a more realistic approach, since a huge variety of microbial metabolites are formed in the colon from flavan-3-ols and from other polyphenols, and are therefore present in the organism together and not isolated.

Furthermore, the bioactivity of not only the microbial metabolites, but also of their conjugated forms has been examined in several studies [21,28,42]. This is of unquestionable relevance, since the microbial catabolites formed in the colon can be further conjugated, and these forms might also contribute to the biological actions of the flavan-3-ols too.

Although the flavan-3-ol colonic metabolic pathway has been almost fully elucidated and many of their microbial metabolites have been identified, their formation has been shown to differ among individuals, as reviewed elsewhere [4]. These interindividual differences in the formation of microbial metabolites can be explained by differences in gut microbiota composition, and this is commonly termed in the literature as “metabotype”. Since several biological properties have been associated with flavan-3-ol microbial metabolites, specific metabotypes producing these catabolites could be predictors of their health effects, as previously shown for other polyphenols [86,87,88].

While metabotypes for determined microbial metabolites from polyphenols, such as ellagitannins [89] and isoflavones [90], have been well studied and described, less is known about the gut metabolic phenotypes that are involved in the production of flavan-3-ol colonic metabolites.

Three metabotypes in the formation of flavan-3-ol microbial metabolites were recently proposed for the first time in a study by Mena et al. (*n* = 11). These were defined by the different production amounts of trihydroxyphenyl-γ-valerolactones, dihydroxyphenyl-γ-valerolactones, and hydroxyphenylpropionic acid [91]. In contrast, another study showed that there were no specific proanthocyanidin-derived microbial metabolites that enabled the stratification of individuals by metabotypes, which suggests no influence of interindividual gut microbiota differences on the diverging health effects of proanthocyanidins [92]. Due to the high number of subjects needed for the identification of metabotypes and the difficulty of using phenyl-γ-valerolactones as determinant for metabotypes [93], more research is needed in this area.

## 5. Conclusions

There are numerous available studies that suggest positive effects of flavan-3-ol microbial metabolites. However, caution needs to be taken when interpreting their results and extrapolating them to humans, since only cellular and animal models have been performed. In this regard, human in vivo studies of microbial metabolites may not be realistic either, since their oral administration by human beings would not reproduce their actual formation through microbial metabolism in the colon. Moreover, other factors need to be considered when evaluating the positive health effects of microbial metabolites, such as individual differences in their colonic production, bioavailability, and concentration in biological fluids and tissues.

Although the interest in microbial metabolites of flavan-3-ols has been growing over the last few years and many studies have been performed with the aim of understanding their biological effects in organisms, further research that clarifies their metabotype-dependent formation, their conjugation, and their pharmacokinetics patterns are needed in an attempt to harmonize the study methodologies and, ultimately, to elucidate their health benefits in human beings.

## Figures and Tables

**Table 1:** Biological activity of phenyl-γ-valerolactones and phenylvaleric acids.^1^

Test/Model	Microbial Metabolite	Concentration/Dose	Results	Ref.
Anti-adhesive activity				
Adherence of uropathogenic *Escherichia coli* (UPEC) to T24 bladder epithelial cells	(*R*)-5-(3’,4’-dihydroxyphenyl)-γ-VL; (*R*)-5-phenyl-γ-VL-3’,4’-di-*O*-sulphate; (*R*)-5-(4’-hydroxyphenyl)-γ-VL-3’-*O*-sulphate; (*R*)-5-(3’-Hydroxyphenyl)-γ-VL-4’-*O*-sulphate	50–100 µM	All metabolites inhibited adherence of UPEC at 100 µM. (*R*)-5-(3’-hydroxyphenyl)-γ-VL-4’-*O*-sulphate also inhibited the adhesion at 50 µM.	[21]
Anti-inflammatory activity				
NO formation and iNOS expression in LPS-exposed RAW 264.7 macrophage; binding to RAW 264.7, EA.hy 926 endothelial cell and human monocyte	5-(3’,4’-dihydroxyphenyl)-γ-VL	IC_50_ = 1.3–3.8 µg/mL	NO production and iNOS expression were inhibited in a concentration-dependent manner. High binding capacity to RAW 264.7, EA.hy 926 and human monocytes, which was reduced in the presence of phloretin.	[22]
Cardiovascular protective effect				
Systolic blood pressure in spontaneously hypertensive rats and ACE activity	5-(3,4,5-trihydroxyphenyl)-γ-VL; 5-(3,5-dihydroxyphenyl)-γ-VL; 4-hydroxy-5-(3,4,5-trihydroxyphenyl)ValA; 4-hydroxy-5-(3,5-dihydroxyphenyl)-ValA; 5-(3,4,5-trihydroxyphenyl)ValA; 5-(3,5-dihydroxyphenyl)ValA; 5-(3-hydroxyphenyl)ValA	150–200 mg/kg; IC_50_ = 1.51–19.59 µM	Systolic blood pressure decreased 2 h after 150 mg/kg 5-(3,4,5-trihydroxyphenyl)-γ-VL intake, and 4 h after 200 mg/kg 5-(3,5-dihydroxyphenyl)-γ-VL. The order of ACE inhibitory activity was: EGCG > 5-(3,4,5-trihydroxyphenyl)ValA > 5-(3,5-dihydroxyphenyl)ValA > 5-(3,4,5-trihydroxyphenyl)-γ-VL ≅ 5-(3-hydroxyphenyl)ValA > EC > 4-hydroxy-5-(3,4,5-trihydroxyphenyl)ValA >> 4-hydroxy-5-(3,5-dihydroxyphenyl)-ValA >> 5-(3,5-dihydroxyphenyl)-γ-VL.	[23]
THP-1 monocyte adhesion to TNF-α-stimulated human umbilical vein endothelial cells	5-(3’,4’-dihydroxyphenyl)-γ-VL	7.5–30 µM	The endothelial adhesion was prevented. Downregulation of VCAM-1 and MCP-1 expression, as well as of NF-κB promoter activity and IKK and IκBα phosphorylation.	[24]
Chemopreventive effect				
Proliferation of human cervical cancer cell (HeLa)	4-hydroxy-5-(3,5-dihydroxyphenyl)VA; 5-(3,5-dihydroxyphenyl)-γ-VL; 4-hydroxy-5-(3,4,5- trihydroxyphenyl)ValA; 5-(3,4,5-tri-hydroxyphenyl)-γ-VL; 5-(3,4,5-trihydroxy-phenyl)ValA; 5-(3,5-dihydroxyphenyl)ValA; 5-(3-hydroxyphenyl)ValA; 4-hydroxy-5-(3,4-dihydroxyphenyl)ValA; 5-(3,4-dihydroxyphenyl)ValA	50 µg/mL	4-hydroxy-5-(3,4,5-trihydroxyphenyl)ValA, 5-(3,4,5-trihydroxyphenyl)ValA and 5-(3,4-dihydroxyphenyl)ValA inhibited the proliferation of HeLa cells by 71.9%, 13.5% and 53.9%, respectively (relative to negative control set at 100, DMSO). 5-(3,4,5-trihydroxyphenyl)ValA had the strongest inhibitory activity among the metabolites (IC_50_ = 5.58 µM).	[25]
Proliferation of androgen-dependent human prostate cancer cells (LNCaP)	5-(3’,4’,5’-trihydroxyphenyl)-γ-VL	IC_50_ = 117 μM	Inhibition of LNCaP proliferation. DHT-induced nuclear translocation of AR was inhibited in 54.5 ± 4.7% of cells.	[26]
Immunomodulatory activity				
NK cell cytotoxicity against murine lymphoma YAC-1 target cells in mouse splenocytes treated in vivo; activation of mice splenic CD4⁺ T cells	5-(3’,5’-dihydroxyphenyl)-γ-VL; 4-hydroxy-5-(3’,5’-dihydroxyphenyl)ValA; 4-hydroxy-5-(3’,4’,5’-trihydroxy-phenyl)ValA; 5-(3’,4’,5’-trihydroxyphenyl)-γ-VL; 5-(3’,5’-dihydroxyphenyl)ValA; 5-(3’,4’,5’-trihydroxyphenyl)ValA and 5-(3’-hydroxyphenyl)ValA	10 mg/kg; 10 µM	NK cell cytotoxic activity increased in the 5-(3’,5’-dihydroxyphenyl)-γ-VL intake group. IFN-γ production was also dose-dependently increased. The order of CD4⁺ T cell activity (ATP) was: 5-(3’-hydroxyphenyl)ValA > 4-hydroxy-5-(3’,5’-dihydroxyphenyl)ValA = 5-(3’,5’-dihydroxyphenyl)-γ-VL > 5-(3’,5’-dihydroxyphenyl)ValA > 4’-dehydroxylated EGC.	[27]
Neuroprotective effect				
Human SH-SY5Y neuroblastoma cells growth and neurite outgrowth	5-(3’,5’-dihydroxyphenyl)-γ-VL and its conjugated forms (glucuronide and sulfate forms)	0.05 µM	5-(3’,5’-dihydroxyphenyl)-γ-VL enhanced SH-SY5Y cell number. Neurite length and number was significantly increased by 5-(3’,5’-dihydroxyphenyl)-γ-VL and its sulfated form. Glucuronide only increased neurite number.	[28]

^1^ ACE: angiotensin I-converting enzyme; CD4⁺ cells: T helper cells; DHT: dihydrotestosterone; DMSO: dimethyl sulfoxide; IFNγ: interferon gamma; IκBα: NF-κB inhibitor α; IC_50_: half maximal inhibitory concentration; IKK: IκB kinase; iNOS: inducible nitric oxide synthase; LPS: lipopolysaccharide; MCP-1: monocyte chemoattractant protein 1; NF-κB: nuclear factor kappa-light-chain-enhancer of activated B cells; NK cells: natural killer cells; NO: nitric oxide; TNF-α: tumor necrosis factor; VCAM: vascular cell adhesion molecule; Flavan-3-ols and microbial metabolites. EGC: (−)-epigallocatechin; ValA: valeric acid; VL: valerolactone.

**Table 2:** Biological activity of phenolic acids.^1^

Test/Model	Microbial Metabolite	Concentration/Dose	Results	Ref.
Anti-adhesive activity				
Adherence of uropathogenic *E. coli* to T24 epithelial bladder cells	Catechol; BA; 3-HB; PCA; VA; GA; PA; 3-HPA; 3,4-DHPA; 3-PP; 3-HPP and 3,4-DHPP	100–500 µM	Catechol, BA, VA, PA and 3,4-DHPA inhibited *E. coli* adherence in a concentration-dependent manner. GA and PA had the strongest effect, followed by 3,4-DHPA.	[32]
Antidiabetic effects				
Glucose transport in human and murine 3T3-L1 adipocytes stimulated or not with insulin	PCA	100 µmol/L	PCA reversed the oxLDL-induced drop in glucose uptake and GLUT4 translocation. PCA also prevented the oxLDL-induced reduction of adiponectin mRNA expression and secretion, as well of PPARγ mRNA expression and activity.	[33]
Beta cell function of rat INS-1E pancreatic beta cells and isolated rat pancreatic islets	3,4-DHPA; 2,3-DHB and 3-HPP	1–5 µM	3,4-DHPA and 3-HPP significantly increased glucose-induced insulin secretion (5 and 1 µM, respectively). In presence of oxidative stress, 3,4-DHPA and 3-HPP reduced ROS and carbonyl group production, and glucose-stimulated insulin secretion was restored to control levels. The phosphorylation of PKC and ERKs was enhanced.	[34]
Beta cell function of Min6 pancreatic beta cells incubated with cholesterol	3,4-DHPA	10–250 µM	3,4-DHPA prevented impaired insulin secretion induced by cholesterol by protecting pancreatic beta cells against oxidative stress, apoptosis and mitochondrial dysfunction.	[35]
Insulin signalling and glucose uptake and production in rat renal NRK-52E cells	2,3-DHB; 3,4-DHPA; 3-HPP and VA	20 µM	Glucose uptake and production decreased after treatment with 2,3-DHB, and PEPCK levels as well. IR and IRS-1 phosphorylated and total protein levels were increased. The inhibition of the PI3K/Akt pathway was restrained.	[36]
Insulin signalling and glucose uptake and production in rat renal NRK-52E cells treated with high glucose	3,4-DHPA; 2,3-DHB and 3-HPP	10 µM	3,4-DHPA restored the altered glucose uptake and production caused by high glucose, and tyrosine phosphorylated and total levels of IR increased. The PI3K/Akt pathway and AMPK were activated, while the PEPCK expression was decreased.	[37]
Beta cell function and glucose utilization in human skeletal muscle and rat INS-1 beta cells	HA; HVA and 5-PVA	5–100 µM	HA and 5-PVA stimulated glucose oxidation in skeletal muscle and preserved skeletal mitochondrial function after oxidative insult. In beta cells, all metabolites induced glucose-stimulated insulin secretion without affecting beta cell mitochondrial respiration or electron transport chain components’ expression.	[38]
Antiglycative activity				
Formation of AGEs in BSA/glucose system and glyoxal trapping ability	PG; 3,4-DHPP; DHFA; 3-HPA; 3,4-DHPA and HVA	2.0–50 µmol/L	Only DHFA at 10 µmol/L had a significant impact inhibiting albumin glycation, and a combination of 3-HPA, 3,4-DHPA and HVA inhibited glycation at 2.0 µmol/L. PG, 3,4-DHPP and 3,4-DHPA showed a glyoxal trapping ability of approximately 60%, 90% and 65%, respectively.	[39]
Formation of AGEs in BSA/glucose and BSA/MGO systems	3,4-DHPA; 3-HPA and HVA	1 mM	The order of inhibitory activity against AGEs was: rutin > quercetin > 3,4-DHPA > aminoquanidine > 3-HPA > HVA	[40]
Anti-inflammatory activity				
NO production in LPS-activated RAW264.7 cells	3-HPP; CA and 3,4-DHPP	IC_50_ = 224.85–689.91 µM	CA and 3,4-DHPP inhibited the NO production significantly stronger than 3-HPP.	[41]
Inflammatory response in LPS-stimulated human THP-1 monocytic cells	4-HBA; BA-glucuronide; BA-sulfate; PCA; PCA-3-glucuronide; PCA-4-glucuronide; PCA-3-sulfate; PCA-4-sulfate; VA; VA-glucuronide and VA-sulfate	0.1–10 µM	LPS-induced TNF-α secretion was inhibited by BA-sulfate, VA-glucuronide and PCA-3-sulfate, as well as by four combinations of metabolites that included 4-HBA and/or PCA with a stronger effect than the individual metabolites. 4-HBA significantly reduced IL-1ß secretion.	[42]
Inflammatory response in LPS-stimulated BV2 microglia	PCA	5–20 µM	PCA dose-dependently inhibited LPS-induced TNF-α, IL-6, IL-1ß and PGE_2_ production, and suppressed LPS-induced TLR4 expression, NF-κB and MAPKs activation.	[43]
Inflammatory response in LPS-stimulated human gingival fibroblasts	PCA	5–20 µM	PCA inhibited LPS-induced IL-6 and IL-8 production and NF-κB activation. PPAR-γ antagonist GW9662 reversed the prevention of IL-6 and IL-8 production by PCA.	[44]
Colitis mice model induced by 2,4,6-trinitrobenzenesulfonic acid (TNBS)	PCA	30 and 60 mg/kg	PCA improved TNBS-induced colitis in mice, reduced the GSSG/GSH ratio and expression of proinflammatory cytokines, and increased the expression of antioxidant enzymes and Nrf2. The SphK/S1P axis and the related NF-κB and STAT3 signaling pathway were abrogated.	[45]
NO production in LPS-Stimulated RAW 264.7 macrophages and dendritic D2SC/I cells	*p*-CoA; HVA; 4-HB; HA; FA; PCA; CA; VA; 3-HPA; 3,4-DHPA	0.1–100 µM	CA, 3,4-DHPA and PCA were the most active metabolites inhibiting NO production in RAW 264.7 cells. In D2SC/I cells, 3,4-DHPA, CA, and *p*-CoA were the most potent metabolites.	[46]
Inflammatory response of HIEC-6 human intestinal epithelial cells after IL-1β-induced ulcerative colitis, and of mice after TNBS-induced ulcerative colitis	GA	20–60 mg/kg	Anti-inflammatory cytokines (IL-4 and IL-10) increased and the proinflammatory ones (IL-1, IL-6, IL-12, IL-17, IL-23, TGF-β and TNF-α) decreased in HIEC-6 cells and in mice. Apoptosis was reduced in GA treated groups and the colonic inflammation in mice was attenuated. GA inhibited NF-κB activation.	[47]
Antioxidant activity				
DPPH radical scavenging activity	3-HPP; CA and 3,4-DHPP	IC_50_ = 5.02–5.91 µM	CA and 3,4-DHPP had the stronger scavenging radical activity, while 3-HPP had no antioxidant activity.	[41]
ABTS assay	4-HPA; 3,4-DHPA; PCA; 2,3-DHB; PG and GA	IC_50_ = 4.332–852.713 µM	The ability to scavenge 50% of free radical ABTS• + was stronger for GA, PG and 3,4-DHPA but weaker for 4-HPA.	[48]
Ferric-reducing antioxidant potential (FRAP)	4-HPA; 3,4-DHPA; PCA; 2,3-DHB; PG and GA	1.00 x 10^-3^ mg/mL	The strongest antioxidant activity was shown by 3,4-DHPA, PG, GA and PCA.	[48]
DPPH radical scavenging activity	3,4-DHPA; 3-HPA and HVA	1 mM	The order of antioxidant activity was: quercetin > rutin = 3,4-DHPA > HVA >> 3-HPA.	[40]
Ferric-reducing antioxidant potential (FRAP)	3,4-DHPA; 3-HPA and HVA	1 mM	The order of reducing activity was: quercetin > HVA > 3,4-DHPA > rutin >> 3-HPA.	[40]
Cyclic voltammetry (CV)	3,4-DHPA; 3-HPA and HVA	1 mM	The order of reducing activity was: quercetin > rutin > 3,4-DHPA > HVA > 3-HPA.	[40]
Ferrozine assay	3,4-DHPA; 3-HPA and HVA	1 mM	The order of chelating activity was: rutin > quercetin > HVA >> 3-HPA >> 3,4-DHPA.	[40]
DPPH radical scavenging assay	3,4-DHPA; 3-HPA; PCA and HA	2–10 µM	The order of antioxidant activity was: 3,4-DHPA = quercetin > PCA > 3-HPA ≅ HA.	[49]
Superoxide scavenging assay	3,4-DHPA; 3-HPA; PCA and HA	50 µM	The order of superoxide scavenging activity was: quercetin > 3,4-DHPA > PCA >> 3-HPA ≅ HA.	[49]
DPPH radical scavenging assay	3,4-DHPA; *p*-CoA; VA and FA	25 µM	The order of antioxidant activity was: 3,4-DHPA > VA > FA > *p*-CoA.	[50]
Ferric-reducing antioxidant potential (FRAP)	3,4-DHPA; *p*-CoA; VA and FA	5 µM	The order of reducing activity was: 3,4-DHPA > VA > FA > *p*-CoA.	[50]
ABTS assay	3,4-DHPA; *p*-CoA; VA and FA	5 µM	The order of antioxidant activity was: 3,4-DHPA > FA > *p*-CoA > VA.	[50]
ORAC assay	3,4-DHPA; *p*-CoA; VA and FA	3 µM	The order of antioxidant activity was: *p*-CoA > 3,4-DHPA > VA > FA.	[50]
Anti-proliferative activity and cytotoxicity				
Apoptosis and cellular oxidative stress of oxLDL-exposed J774A.1 cells	PCA	25 µM	OxLDL-induced cell death was prevented, as well as ROS production and GSH depletion. The activation of p53 was prevented, and therefore the overexpression of p53-target genes decreased. p38MAPK and PKC∂ activation was reversed. PCA induced JNK activation and increased nuclear Nrf2 content.	[51]
TGF-ß1-induced proliferation and migration of human airway smooth muscle cells (ASMCc)	PCA	1–50 nM	PCA inhibited the proliferation and migration of ASMCs and the expression of type I collagen and fibronectin. The Smad2/3 activation in ASMCs exposed to TGF-ß1 was downregulated.	[52]
Cardiovascular protective effect				
Antithrombotic efficacy under high shear stress in vitro in human platelets as well as in an in vivo arterial thrombosis model	PCA	5–25 µM	PCA significantly decreased stress-induced platelet aggregation by blocking the interaction between von Willebrand factor (vWF) and glycoprotein Ib. Intracellular calcium increase was attenuated, shear-induced granular secretion from dense and α-granules was inhibited and glycoprotein IIb/IIIa activation was attenuated. The antithrombotic effects of PCA were confirmed in vivo.	[53]
Cardiac function and cardiac autonomic balance in STZ-induced diabetic rats	PCA	50 and 100 mg/kg	%FS and %LVEF increased and LF:HF decreased compared with untreated diabetic rats. Plasma HbA1c decreased as well as cardiac MDA and cardiac mitochondrial ROS. Mitochon-drial membrane depolarization and swelling was prevented and cardiac anti-apoptotic BCL2 protein levels increased	[54]
Vasodilation of pre-contracted isolated aortic rings; blood pressure in normotensive and spontaneously hypertensive rats	3-PP; 4-HPP; 3,4-DHPP; 4-HPA; 3,4-DHPA; HVA; 3-HB; PhG; 4-MC; *m*-CoA; 3-HPP and 3-HPA	100 nM; 2.5–25 mg/kg and 5 mg/kg/50 µL/min	3-HPP had the highest vasodilatory activity, which was NO and endothelium-dependent. In vivo, 3-HPP lowered arterial blood pressure in normotensive and spontaneously hypertensive rats.	[55]
Diabetic cardiomyopathy in type 2 diabetic rats	PCA	50 and 100 mg/kg	PCA was protective against diabetic cardiomyopathy through hypoglycemic, insulin-sensitizing, anti-inflammatory and antioxidant effects.	[56]
Insulin-stimulated NO production by human aortic endothelial cells under high glucose conditions	3-HPP	1 µM	Under glucotoxic conditions, 3-HPP preserved insulin-stimulated increases in NO production, and phosphorylation of Akt and eNOS. The rise in ROS and RNS was prevented.	[57]
Endothelial function and oxidative stress in human Ea.hy926 endothelial cells	3,4-DHPA; 2,3-DHB and 3-HPP	10–12 µM	3,4-DHPA and a mix of the metabolites increased the NO generation and phosphorylation of eNOS, Akt and AMPK. Under oxidative stress, metabolites enhanced cell viability and prevented reduced eNOS phosphorylation. ROS generation and phosphorylation of ERK and JNK were prevented.	[58]
Relaxation of pre-contracted rat artery rings and blood pressure in spontaneously hypertensive rats	3,4-DHPA; 4-MC and 3-HPP	EC_50_ = 22.4–49.1 µM; 0.5–25 mg/kg and 5 mg/kg/min	The vasorelaxant activity of 3,4-DHPA and 4-MC was similar in aorta and mesenteric artery. The effect of 3,4-DHPA depended on endothelium, NO, prostaglandin and Ca^2+^-activa-ted K^+^ channels. Both metabolites dose-dependently decreased blood pressure after bolus and infusion administration.	[59]
Whole blood platelet aggregation induced by arachidonic acid and *ex ovo* hen’s egg model of thrombosis	4-MC and PG	IC_50_ = 3–25 µM; 5 mM	PG showed a comparable anti-platelet effect to that of acetylsalicylic acid, while that of 4-MC was significantly lower. 4-MC interfered with calcium intracellular signalling, being this the possible mechanism of action. In the *ex vivo* experiment, the anti-platelet effect of 4-MC was confirmed by significantly increasing the survival of the eggs.	[60]
Chemopreventive effect				
ATP production by HCT-116 colon cancer cells	3,4-DHPA; 3-HPA; 4-HPA; HVA and 3-OMGA	IC_50_ = 260 µmol/L	3-OMGA inhibited cell proliferation. Combining 3,4-DHPA (100, 200 and 300 µmol/L) and EGCG (40 µmol/L) increased the anti-proliferative effect compared to individual treatments.	[61]
Apoptosis of HT-29 colon cancer cells	4-HPA and VA	100 µM	4-HPA enhanced the late-stage apoptosis and the percentage of dead cells compared to control cells.	[62]
Angiogenesis in HUVEC cells treated with VEGF and in zebrafish model	PCA	6.25–100 μM; 25 µM	PCA inhibited the proliferation, migration, invasion and capillary structure formation of HUVECs, and blocked the VEGFR2-dependent Akt/MMP2 and ERK pathways. In vivo, the anti-angiogenic effect of PCA was possibly due to downregulation of VEGFα-VEGFR2 and Ang2-Tie2 pathways.	[63]
Proliferation and apoptosis of HT-29 colon cancer cells	3,4-DHPA; *p*-CoA; VA and FA	0.1–100 µM	3,4-DHPA had the strongest effect reducing cell viability. All metabolites reduced cell number in S phase, and *p*-CoA and FA increased apoptosis.	[50]
Proliferation, cell-cycle arrest and apoptosis of Caco-2 cell	CA; 3-PP and BA	100–1000 µM and EC_50_ = 460–500 µM	Only CA reduced cell proliferation by 50%, while 3-PPA and BA decreased it at 1000 μM. CA and 3-PP induced cell-cycle arrest at the S-phase. CA activated caspase-3 and 3-PPA decreased mitochondrial DNA content.	[64]
Apoptosis and autophagy in OVCAR-3 ovarian cancer cells	PCA	5–30 µM	PCA inhibited cell proliferation by inducing apoptosis and autophagy. PCA modulated proapoptotic and anti-apoptotic proteins (Bax, Bcl-2, PARP and caspase-3) and upregulated autophagy-related protein LC3-II.	[65]
Modulation of drug metabolizing enzymes				
GSTT2 and COX-2 expression in LT97 human colorectal adenoma cells	3,4-DHPA and 3,4-DHPP	2.5–25 µM	GSTT2 mRNA expression was enhanced up to 1.8-fold. COX-2 mRNA and protein expression were reduced, as well as CumOOH-induced DNA damage.	[66]
Gene expression of drug-metabolizing enzymes in Hepa1c1c7 mouse hepatoma cells	3,4-DHPA; 3-HPA; PCA and HA	5–250 µM	3,4-DHPA increased GCLC, HO-1, NQO1, xCT and CYP1A1 gene expression. PCA at 50 µM increased the gene expression of NQO1. Peroxide-induced cytotoxicity was inhibited.	[49]
Acetaminophen-induced liver injury in mice	3,4-DHPA	10–50 mg/kg	Acetaminophen-induced hepatotoxicity was attenuated by 3,4-DHPA. Nrf-2 translocation to the nucleus was increased, as well as the expression of phase-II (UGT, SULT, GCLC and GCLM) and antioxidant enzymes.	[67]
ALDH activity and gene expression in Hepa1c1c7 mouse hepatoma cells	3,4-DHPA	5–50 µM	Concentration-dependent enhancement of the ALDH activity, as well as expression of ALDH1A1, ALDH2 and ALDH3A. Nuclear levels of Nrf2 and AhR increased significantly at 20 µM, while those of NF-κB decreased.	[68]
Menadione-induced liver damage in rats	PCA	10 and 20 mg/kg	PCA prevented menadione mediated-alterations in hepatocellular markers and it also increased the activities of antioxidant enzymes (SOD and CAT) and phase II detoxifying enzymes (GST and NQO-1), and Nrf-2.	[69]
Modulation of intestinal microbiota				
Alteration of the composition in fecal microbiota of ApoE-/- mice fed on a high-fat diet	FA	30 mg/kg	FA significantly lowered the ratio of *Firmicutes* to *Bacteroidetes* when compared to obese control group.	[70]
Growth inhibition of pathogenic and probiotic bacteria	GA; VA; FA and PCA	MIC = 20–35 mmol/L MBC = 20–30 mmol/L	MIC against *E.coli* and *Staphylococcus* Typhimurium was similar among metabolites (15-20 mmol/L). VA and PCA had the lowest MBC (20 mmol/L). *Lactobacillus acidophilus* and *L. rhamnosus* were also inhibited but at higher concentrations (MIC > 35 mmol/L) and their MBC was also > 35 mmol/L.	[71]
Modulation of lipid metabolism				
Lipid metabolism in HFD-induced obesity in mice	FA	25 and 50 mg/kg	FA reduced serum TC, TG and NEFA levels as compared to the obese control mice. Liver TC and TG significantly decreased as well. SREBP1c, FAS and ACC were reduced, while CPT1a and PPARα were up-regulated.	[72]
Lipid metabolism in HFD-induced obesity in mice	FA	30 mg/kg	Serum TC, TG and LDL-C decreased when compared to obese control group, and liver TC and TG levels as well. FA significantly increased the mRNA expression of AHR and decreased that of FAS and SREBP-1c.	[70]
Neuroprotective effect				
Human neuroblastoma SK-N-MC cell viability after DMNQ-induced oxidative stress	PG; 3,4-DHPP; DHFA; 3-HPA; 3,4-DHPA and HVA	0.1–20 µmol/L	The greatest increase in cell survival was induced by DHFA, followed by PG and 3,4-DHPA. Combination of metabolites also increased cell survival after oxidative stress.	[39]
Type 2 diabetes-induced neurodegeneration in rats	GA and *p*-CoA	20–40 mg/kg	Treatment of diabetic rats with GA and *p*-CoA enhanced the histology of the hippocampus and glucose tolerance, prevented brain oxidative stress, improved antioxidant status, reduced inflammation and inhibited apoptosis.	[73]
Neuroinflammation model based on SIN-1 stress-induced injury in human SH-SY5Y neuroblastoma cells	3,4-DHPA; 3-HPP and 3-HPA	0.1–10 µM	Metabolites increased cell viability, probably through inhibition of ERK1/2, modulation of p38 MAPK kinases (3-HPA), and reduction of caspase-3 activation (3-HPP).	[74]
Apoptosis of human SH-SY5Y neuroblastoma cells previous or during H_2_O_2_ exposition.	3,4-DHPP; 3,4-DHPA and GA	5–10 µM	All metabolites decreased late apoptosis, but 3,4-DHPP had the strongest effect. ROS levels decreased and REDOX activity increased. All metabolites attenuated H_2_O_2_-induced activation of caspases-3 and -9.	[75]
Apoptosis of rat cerebellar granule neurons under H_2_O_2_-induced oxidative stress, nitrosative stress and excitotoxicity	4-HBA and PCA	10–300 µM	Both 4-HBA and PCA mitigated oxidative stress induced by H_2_O_2_. Under conditions of nitrosative stress only PCA was neuroprotective, but under conditions of excitotoxicity only 4-HBA reduced cell death.	[76]
Ischemia-induced hippocampal neuronal death in rats	PCA	30 mg/kg	PCA decreased neuronal cell death, oxidative stress, microglial activation, astrocyte activation and BBB disruption compared with the control group after ischemia. GSH glutathione reduced concentration was recovered.	[77]
Lysolecithin (LPC)-induced model of inflammation in mouse hippocampal neurons co-cultured with glial cells	GA and VA	0.2–1 µM	GA and VA increased neurite outgrowth and upregulated myelin protein in neurites and oligodendrocyte cell bodies. COX-2, NFκB, TN-C, CSPGs and GFAP expression in astrocytes decreased. GA and VA reversed the reduction in sustained repetitive firing induced by LPC.	[78]
Osteoprotective effects				
Osteoclast differentiation and function in mouse bone marrow macrophages treated with RANKL; inflammatory bone destruction in LPS-treated mice	PCA	25 µM; 25 mg/kg	PCA inhibited osteoclastogenesis and the bone-resorbing activity of mature osteoclasts. LPS-mediated bone loss in vivo was also restored by PCA.	[79]
Osteoclast differentiation and apoptosis in RAW264.7 murine macrophage cells treated with RANKL	PCA	8 µM	PCA inhibited osteoclast differentiation by regulating oxidative stress and inflammation, and induced apoptosis in mature osteoclasts by inducing mitochondrial membrane potential, Cyt c release and caspase activation.	[80]
Renoprotective effects				
Redox status in high-glucose-exposed rat renal proximal tubular NRK-52E cell	3,4-DHPA	10 µM	3,4-DHPA reversed the increase in ROS levels and the decreased antioxidant defense. SIRT-1 increased, and the high glucose-induced increase of phosphorylated MAPKs and NOX-4 were restored.	[81]
Extracellular matrix accumulation in high glucose-induced human mesangial cells	PCA	5 and 10 µM	PCA inhibited high glucose-induced proliferation of mesangial cells and protected them against high glucose damage inhibiting the p38 MAPK signaling pathway.	[82]

^1^ABTS: 2-azinobis-(3-ethylbenzothiazoline-6-sulphonate) radical cation; AGE: advanced glycation end-products; Akt: protein kinase B; ALDH: aldehyde dehydrogenase; AMPK: adenosine monophosphate-activated protein kinase; Ang2: angiotensin-2; AR: androgen receptor; BBB: blood-brain barrier; Bcl-2: B-cell lymphoma 2; BSA: bovine serum albumin; CAT: catalase; COX-2: cyclooxygenase-2; CSPG: chondroitin sulfate proteoglycans; CYP1A1: cytochrome P450 1A1; DMNQ: 2,3-dimethoxy-1,4-naphtoquinone; DMSO: dimethyl sulfoxide; DPPH: 2,2-diphenyl-1-picrylhydrazyl radical; EC_50_: half maximal effective concentration; eNOS: endothelial nitric oxide synthase; ERK: extracellular signal–regulated kinases; FS: fractional shortening; GCLC: glutamate-cysteine ligase catalytic subunit; GCLM: glutamate-cysteine ligase modifier subunit; GFAP: glial fibrillary acidic protein; GLUT: glucose transporter; GPx: glutathione peroxidase; GSH: glutathione; GSIS: glucose-stimulated insulin secretion; GSK-3: glycogen synthase kinase-3; GST: glutathione S-transferase; GSTT2: glutathione S-transferase theta-2; HO-1: heme oxygenase 1; HUVEC: human umbilical vein endothelial cells; IC_50_: half maximal inhibitory concentration; IL: interleukin; iNOS: inducible nitric oxide synthase; IR: insulin receptor; IRS-1: insulin receptor substrate 1; JNK: c-Jun N-terminal kinases; LC3: microtubule-associated proteins 1A/1B light chain 3B; LF:HF: low-frequency:high-frequency; LPC: lysolecithin; LPS: lipopolysaccharide; LVEF: left ventricular ejection fraction; MAPK: mitogen-activated protein kinase; MBC: minimal bactericidal concentration; MCP-1: monocyte chemoattractant protein 1; MGO: methylglyoxal; MIC: minimal inhibitory concentration; MMP2: matrix metalloproteinase-2; NF-κB: nuclear factor kappa-light-chain-enhancer of activated B cells; NK cells: natural killer cells; NO: nitric oxide; NOX-4: NADPH-oxidase-4; NQO1: NADH:quinone oxidoreductase-1; Nrf2: nuclear factor (erythroid-derived 2)-like 2; ORAC: oxygen radical absorbance capacity; oxLDL: oxidized LDL; PARP: poly (ADP-ribose) polymerase; PI3K: phosphatidylinositol-3-kinase; PEPCK: phosphoenolpyruvate carboxykinase; PGE_2_: prostaglandin E_2_; PKC: protein kinase C; PPARγ: peroxisome proliferator-activated receptor-γ; RANKL: receptor activator for nuclear factor κB ligand; RNS: reactive nitrogen species; ROS: reactive oxygen species; SGLT-2: sodium-glucose co-transporter-2; SIN-1: 3-morpholinosyndnomine; SIRT-1: sirtuin 1; SOD: superoxide dismutase; SphK/S1P: sphingosine kinase/sphingosine 1-phosphate; STAT-3: signal transducer and activator of transcription 3; STZ: streptozotocin; SULT: sulfotransferase; TGF-ß: transforming growth factor beta; Tie: tyrosine kinase receptor; TLR-4: toll-like receptor 4; TN-C: Tenascin-C; TNFα: tumor necrosis factor; UGT: UDP-glucuronosyltransferase; VCAM: vascular cell adhesion molecule; VEGF: vascular endothelial growth factor; VEGR-2: vascular endothelial growth factor receptor 2; xCT: cystine/glutamate anti-porter. Flavan-3-ols and microbial metabolites. BA: benzoic acid; CA: caffeic acid; DHFA: dihydroferulic acid; EC: (−)-epicatechin; EGC: (−)-epigallocatechin; EGCG: (−)-epigallocatechin gallate; FA: ferulic acid; GA: gallic acid; HA: hippuric acid; HVA: homovanillic acid; *m*-CoA: m-coumaric acid; *p*-CoA: p-coumaric acid; PA: phenylacetic acid; PCA: protocatechuic acid; PG: pyrogallol; PhG: phloroglucinol; VA: vanillic acid; 3-HB: 3-hydroxybenzoic acid; 4-HB:; 3-HPA: 3-hydroxyphenylacetic acid; 4-HPA: 4-hydroxyphenylacetic acid; 3-HPP: 3-hydroxyphenylpropionic acid 4-HPP: 3-(4-hydroxyphenyl)-propionic acid; 4-MC: 4-methylcatechol; 3-OMGA: 3-*O*-methylgallic acid; 3-PP: 3-phenylpropionic acid; 2,3-DHB: 2,3-dihydroxybenzoic acid; 3,4-DHPA: 3,4-dihydroxyphenylacetic acid; 3,4-DHPP: 3,4-dihydroxyphenylpropionic acid; 3,5-DHPP: 3-(3’,5’-dihydroxyphenyl)propionic acid.
